# Essential role for phytol kinase and tocopherol in tolerance to combined light and temperature stress in tomato

**DOI:** 10.1093/jxb/erx356

**Published:** 2017-11-24

**Authors:** Livia Spicher, Juliana Almeida, Katharina Gutbrod, Rosa Pipitone, Peter Dörmann, Gaétan Glauser, Magdalena Rossi, Felix Kessler

**Affiliations:** 1 Laboratory of Plant Physiology, University of Neuchâtel, Switzerland; 2 Departamento de Botânica, Instituto de Biociências, Universidade de São Paulo, Brazil; 3 Institute of Molecular Physiology and Biotechnology of Plants, University of Bonn, Germany; 4 Neuchâtel Platform of Analytical Chemistry, University of Neuchâtel, Switzerland

**Keywords:** High-light stress, high-temperature stress, lipidomics, phytol, phytol kinase, plastochromanol, plastoglobules, plastoquinone, *Solanum lycopersicum*, tocopherol

## Abstract

In a changing environment, plants need to cope with the impact of rising temperatures together with high light intensity. Here, we used lipidomics in the tomato model system to identify lipophilic molecules that enhance tolerance to combined high-temperature and high-light stress. Among several hundred metabolites, the two most strongly up-regulated compounds were α-tocopherol and plastoquinone/plastoquinol. Both are well-known lipid antioxidants and contribute to the protection of photosystem II (PSII) against photodamage under environmental stress. To address the protective function of tocopherol, an RNAi line (*vte5*) with decreased expression of VTE5 and reduced levels of α-tocopherol was selected. VTE5 encodes phytol kinase, which acts in the biosynthetic pathway of tocopherols. *vte5* suffered strong photoinhibition and photobleaching when exposed to combined high-light and high-temperature stress, but neither stress alone produced a visible phenotype. As *vte5* had plastoquinone levels similar to those of the wild type under combined stress, the strong phenotype could be attributed to the lack of α-tocopherol. These findings suggest that VTE5 protects against combined high-light and high-temperature stress and does so by supporting α-tocopherol production.

## Teaser

An essential role is identified for tocopherol in tomato under combined high-light and high-temperature stress. The dual antioxidant and membrane-stabilizing properties of tocopherol may explain its role in combined stress tolerance.

## Introduction

Plants are sessile organisms and are therefore constantly exposed to environmental challenges. Climate change scenarios include high temperatures (HT) that may occur in conjunction with high light (HL) intensity, which together could undermine plant survival and affect agricultural yields (Walther *et al.*, 2002; Streb *et al.*, 2003; Pretty *et al.*, 2010).

Protection of the photosynthetic machinery embedded in the thylakoid membrane is paramount. Plastoquinone (mostly known as an electron transporter) and tocopherols are lipid-soluble molecules that act as antioxidants, preventing lipid peroxidation and photoinhibition by quenching reactive oxygen species (ROS) under HL intensities (Munné-Bosch and Alegre, 2002; Gruszka *et al.*, 2008; Triantaphylidès and Havaux, 2009; Mène-Saffrané and DellaPenna, 2010; Nowicka and Kruk, 2012; Rastogi *et al.*, 2014; Ksas *et al.*, 2015; Miret and Munné-Bosch, 2015).

Tocopherols (collectively known as vitamin E) contribute to seed longevity, seedling development, and protection of the photosynthetic apparatus against oxidative stress (Sattler *et al.*, 2004; Mène-Saffrané *et al.*, 2010), and their synthesis is increased under these conditions (Collakova and DellaPenna, 2003; Munné-Bosch, 2005; Maeda *et al.*, 2006; Loyola *et al.*, 2012; Eugeni Piller *et al.*, 2012; Quadrana *et al.*, 2013; Spicher *et al.*, 2016). In addition to its function as a lipid antioxidant, α-tocopherol physically stabilizes membrane structures by reducing membrane fluidity (Arora *et al.*, 2000; Wang and Quinn, 2000). Yet, the conditions under which tocopherols are essential are currently not known. The synthesis of tocopherol requires condensation of the aromatic homogentisate ring derived from the plastidial shikimate pathway and a phytol diphosphate chain. The majority of the phytol required is salvaged from chlorophyll catabolism rather than synthesized *de novo* (vom Dorp *et al.*, 2015; Almeida *et al.*, 2016). The salvage pathway requires phytol kinase (VTE5) and phytyl-phosphate kinase (VTE6), converting free phytol into phytyl diphosphate; *vte5* and *vte6* mutant plants accumulate less tocopherol (Valentin *et al.*, 2006; Ischebeck *et al.*, 2006; vom Dorp *et al.*, 2015; Almeida *et al.*, 2016). Apart from tocopherol, phytol may also be converted into fatty acid phytyl esters (FAPEs) (Ischebeck *et al.*, 2006; Gaude *et al.*, 2007; Lippold *et al.*, 2012). This process detoxifies free phytol, which has detergent-like characteristics (Dörmann, 2007).

In this study, we demonstrate that VTE5, through its effect on tocopherol levels, plays an essential role in protection of tomato against combined HT and HL stress.

## Materials and methods

### Plant material

A tomato VTE5-knockdown transgenic line (*SIVTE5*-RNAi#1, here termed *vte5*, background in cv. Micro-Tom; MT) had been generated in a previous study (Almeida *et al.*, 2016) by constitutively expressing an intron-spliced hairpin sequence targeting the *SlVTE5* gene (Solyc03g071720). Wild-type (WT; *Solanum lycopersicum*, cv. MT) and *vte5* plants were grown in soil under conditions referred to as control (250 µmol m^−2^ s^−1^ light, 16/8 h light/dark, at 20/18 °C, with 55% relative air humidity).

### Stress treatments

Plants 5 to 6 weeks old were either kept under standard growth conditions (control, as described above) or subjected for 6 days to stress treatments: HL (800 µmol m^−2^ s^−1^ light, 16/8 h light/dark, at 20/18 °C), HT (250 µmol m^−2^ s^−1^ light, 16/8 h light/dark, at 38/30 °C), and a combination of HL and HT (800 µmol m^−2^ s^−1^ light, 16/8 h light/dark, at 38/30 °C).

### Transmission electron microscopy

Transmission electron microscopy was used to analyze the chloroplast ultrastructure of WT and *vte5* and to compare their ultrastructure under HL, HT, and the combination of HT+HL stress treatments. Leaf segments from tomato plants exposed for 6 continuous days to stress treatments were fixed under vacuum overnight in 0.1 M phosphate buffer (pH 6.8) containing 4% (w/v) formaldehyde and 5% (w/v) glutaraldehyde, washed three times in 0.1 M phosphate buffer (pH 6.8) for 20 min, and postfixed for 2 h with 1% (w/v) osmium tetroxide at 20 °C. Samples were washed in 0.1 M phosphate buffer and then dehydrated in a graded series of ethanol and acetone. Samples were first infiltrated overnight in Spurr resin (Polyscience). Leaf fragments were then placed in an appropriate mold in Spurr resin and heated at 60 °C for 24 h to allow solidification and embedding of the leaf section in the resin. Ultrathin sections of 90 nm were prepared using an Ultracut-E microtome (Reichert-Jung) equipped with a diamond knife (Diatome), mounted on copper grids, and contrasted with a saturated uranyl acetate solution in 50% ethanol (Watson, 1958) and with Reynolds’ lead citrate (Reynolds, 1963). Ultrathin sections were observed with a Philips CM-100 electron microscope operating at 60 kV.

### Determination of photosynthetic parameters

The ratio of maximum photochemical efficiency or optimum quantum yield of PSII (*F*v/*F*m), electron transport rate (ETR), and non-photochemical quenching (NPQ) were fluorometrically determined using a MINI-PAM Photosynthesis Yield Analyzer (Walz). Plants were dark-adapted for 15 min before *F*v/*F*m measurements with illumination by application of a saturation flash. Measurements were always made in the green photosynthetic tissue. At least four replicates for each treatment were performed, at day 0 as a baseline measurement before the application of stress, and at days 2, 4, and 6 after the start of continuous stress treatment. On day 7, all plants were exposed to control conditions in order to assess recovery after stress. Another measurement was taken after 5 days of recovery (day 11).

### Untargeted lipid profiling

After 6 days of the HT, HL, or combined HT+HL treatment, at least four replicates of leaf material from each treatment were harvested for untargeted lipid analysis (Martinis *et al.*, 2013). Lipids were extracted from 100 mg fresh leaf tissue using 1 ml tetrahydrofuran:methanol 50:50 (v/v) according to Spicher *et al.* (2016). After centrifugation, the supernatant was directly analyzed by ultrahigh-pressure liquid chromatography (UPLC^TM^, Waters) coupled to quadrupole time-of-flight mass spectrometry (QTOF-MS; Synapt G2, Waters) through an atmospheric pressure chemical ionization (APCI) interface. To evaluate variations in the WT and *vte5*, features were extracted from lipidomics data using Markerlynx XS (Waters), mean-centered, and Pareto-scaled before applying principal component analysis (PCA) (Martinis *et al.*, 2011; Eugeni Piller *et al.*, 2011). The changes in lipid content after the stress treatments were established after the PCA, reducing data complexity. Identification of the variables of interest was achieved through comparison with pure standards whenever available.

When standards were not available, tentative identification was performed by combining determination of elemental compositions (with accurate mass and isotopic ratios provided by QTOF-MS), fragmentation by collision-induced dissociation to obtain characteristic fragments, and searches in online databases such as LIPID MAPS (http://www.lipidmaps.org/data/structure/LMSDSearch.php?Mode=SetupTextOntologySearch) and PUBCHEM (https:// pubchem.ncbi.nlm.nih.gov/search/search.cgi#).

### Targeted lipid profiling: prenylquinone, carotenoid, and glycolipid profile

Absolute concentrations of δ-tocopherol (δ-T), γ-tocopherol (γ-T), α-tocopherol (α-T), α-tocopherolquinone (α-TQ), plastoquinone (PQ-9), plastochromanol (PC-8), and phylloquinone (vitamin K) were measured based on calibration curves obtained from standards as described by Martinis *et al.* (2013). Plastoquinol (PQH_2_-9), hydroxy-plastoquinone (PQ-OH), and hydroxy-plastochromanol (PC-OH) were quantified as PQ-9 and PC-8 equivalents, respectively. Tocopherol and phylloquinone standards were purchased from Sigma-Aldrich. Pure standards of PQ-9 and PC-8 were obtained by purification from spinach leaves and flaxseed oil, respectively. PQ-9 was extracted from entire leaves in acetone and further purified by normal-phase open-column chromatography followed by reverse-phase semi-preparative HPLC (Malferrari and Francia, 2014). PC-8 was obtained by saponification of the oil followed by partition with a hexane:ethylacetate mixture (Siger *et al.*, 2014). The resulting extract was then separated by open-column chromatography and semi-preparative HPLC as for PQ-9 isolation. A detailed description of the purification process is given in Supplementary Protocol S1 at *JXB* online. The two carotenoids violaxanthin and neoxanthin were measured as a sum, since they could not be resolved in either the chromatographic or the mass dimensions under the conditions employed.

The other molecules identified for which pure standards were unavailable were quantified relatively based on peak intensity measurements in the chromatograms. The data obtained from the measurements were subjected to one-way ANOVA, followed by Holm-Sidak test comparisons *versus* control in WT, to determine any significant differences between different temperatures and light conditions over the time course of the experiment.

### Free phytol and fatty acid phytyl ester quantification

Total lipids were extracted from ~20 mg lyophilized leaf tissue with diethylether and 300 mM ammonium acetate. Next, FAPEs and free phytol were purified via solid-phase extraction on silica columns (Strata Si-1, 100 mg, Phenomenex) using a step gradient of *n*-hexane and diethylether (www.cyberlipid.org): FAPEs were eluted with *n*-hexane:diethylether 99:1 (v/v), while free phytol was eluted with *n*-hexane:diethylether 92:8 (v/v). FAPEs and free phytol were analyzed as previously described (vom Dorp *et al.*, 2015).

### Quantitative real-time PCR analysis

RNA extraction, cDNA synthesis, and quantitative real-time PCR (qPCR) assays were performed as described by Quadrana *et al.* (2013). Specific primers to genes in the tocopherol biosynthetic and related pathways were designed. Primer sequences are listed in Supplementary Table S1. qPCRs were performed in a 7500 real-time PCR system (Applied Biosystems) using 2x SYBR Green Master Mix reagent (Applied Biosystems). Expression values were normalized against the geometric mean of two reference genes, *CAC* and *EXPRESSED* (Quadrana *et al.*, 2013). RNA samples with absorbance ratios at 260/280 nm between 1.95 and 2.1, indicating high purity, and showing sharp and clear rRNA 28S/18S bands on an agarose gel, as an indication of structural integrity, were used for subsequent cDNA synthesis. A permutation test lacking sample distribution assumptions (Pfaffl *et al.*, 2002) was applied to detect statistically significant differences (*P*<0.05) in expression ratios using the algorithms in the fgStatistics software package (Di Rienzo *et al.*, 2009).

### Isolation of thylakoid membranes and Hill reaction

Thylakoid membranes were prepared from 50 g of fresh leaves from 18-day-old pea (*Pisum sativum*) plants, which were grown under control conditions with minor modifications (Fitzpatrick and Keegstra, 2001; Smith *et al.*, 2003; Kummerová *et al.*, 2006, 2008). The Hill reaction was adapted and carried out to measure the direct effect of phytol on the photosynthetic primary reaction at the level of PSII. The total reaction mixture (4 ml) contained 0.03 mM sodium salt solution of 2,6-dichlorophenolindophenol (DCPIP; redox system), 20% dimethyl sulfoxide (DMSO, Sigma-Aldrich) and 0.5 and 1 mg thylakoid membranes in suspension buffer. Phytol (97%, mixture of isomers) was purchased from Sigma-Aldrich and suspended in DMSO at a final concentration of 10 mM. Phytol was used to obtain final concentrations of 500 µM and 50 µM. The reactions were incubated for 5 minutes and then exposed for 6 minutes to an irradiance of 200–250 µmol m^−2^ s^−1^ at room temperature. A 1 ml aliquot was removed at 3 and 6 minutes, centrifuged for 1 min at 16000 *g*, and measured spectrophotometrically at 600 nm. Photosynthetic activity was determined by measuring DCPIP reduction using an UltraViolet-Visible Spectroscopy (UV/VIS) Ultrospec 3100 pro (Amersham Biosciences) in 1 cm UV-visible cuvettes. Measurements were repeated at least three times at each time point.

## Results

### Untargeted lipidomics demonstrate changes in lipid composition upon combined high-light and high-temperature stress

**Fig. 1.**
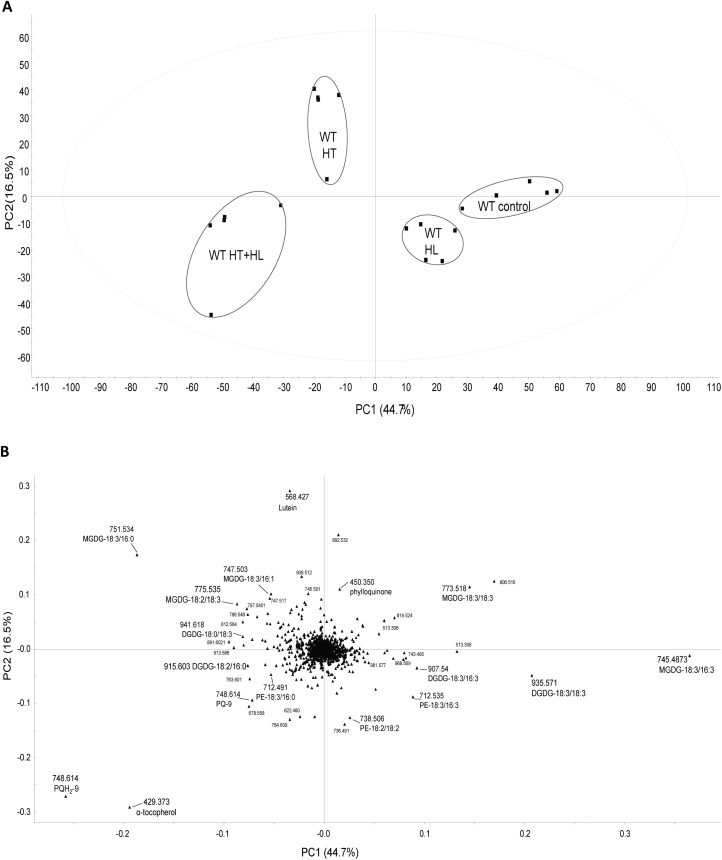
Untargeted lipidomics profile of WT tomato leaves subjected to 6 days of HL, HT, and the combination of HT+HL stress treatments. (A) Principal component analysis (PCA) of the lipid composition of WT leaf samples exposed to HL, HT, and HT+HL for 6 days. PC1 and PC2 are the first and second principal components, respectively, and their percentages of explained variance are shown on the axes. (B) Corresponding loading plots. Data were mean-centered and Pareto-scaled before PCA.

To determine variations in the lipid composition of WT tomato plants after exposure to HL, HT, and the combination of HT+HL, we carried out untargeted lipidomics analysis (Fig. 1). Lipid extracts were isolated from fresh leaf tissue and analyzed by ultrahigh-pressure liquid chromatography coupled with atmospheric pressure chemical ionization-quadrupole time-of-flight mass spectrometry (UHPLC-APCI-QTOF-MS). PCA identified four distinct clusters when comparing the control condition with the different stress conditions (Fig. 1A). In the loadings plot (Fig. 1B), the most contributive features of the first principal component (PC1) were selected and characterized by a combination of tandem mass spectrometry analysis and consultation of databases such as LIPID MAPS. This revealed that prenylquinones, α-tocopherol, and plastoquinol (PQH_2_-9) contributed most to the separation of the HT+HL cluster. The HT cluster was characterized by the accumulation of the saturated galactolipid MGDG-18:3/16:0, and the control and HL clusters were specifically separated from the other clusters, represented by MGDG-18:3/16:3, MGDG-18:3/18:3, and DGDG-18:3/18:3 (Fig. 1B). Loadings located near the center of the plot, (i.e. the vast majority) make a negligible contribution to metabolic variation.

### *vte5* develops a chlorotic phenotype under combined high-light and high-temperature stress

**Fig. 2.**
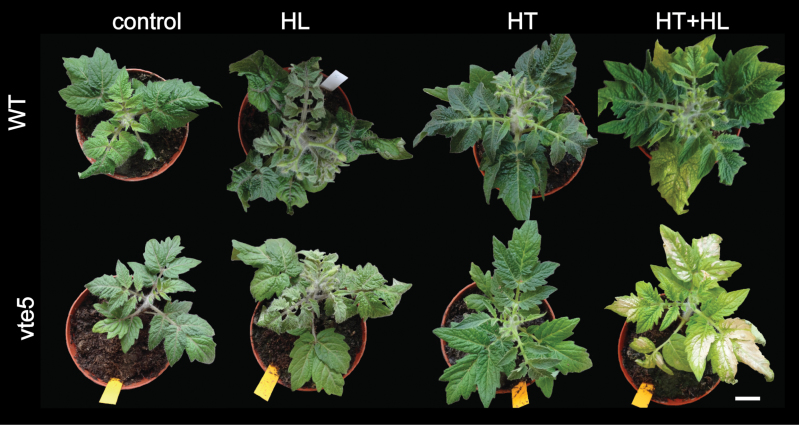
Visible phenotypes of WT and *vte5* after 6 days under control, HL, HT, and combined HT+HL conditions. Bar=3 cm.

To investigate the role of tocopherol in resistance to HT+HL stress, a VTE5-deficient tomato line (*vte5*) was used (Almeida *et al.*, 2016). The *vte5* line contains diminished levels of phytol kinase and therefore accumulates reduced levels of tocopherol in leaves and fruits (Almeida *et al.*, 2016). The line was subjected to the HL, HT, and HT+HL conditions. HL treatment alone had little effect on WT and *vte5* plants. Under HT treatment, no differences were apparent between WT and *vte5* or when compared with control conditions. While WT plants developed a mild bleaching phenotype only in the oldest leaves after 6 days under the HL+HT condition, *vte5* plants were extensively photobleached and developed chlorotic leaves (Fig. 2); the chlorosis became visible after 2 days under HL+HT. The visible phenotype after 4 days is shown in Supplementary Fig. S1.

### Combination of high light and high temperature triggers photoinhibition in *vte5* plants

**Fig. 3.**
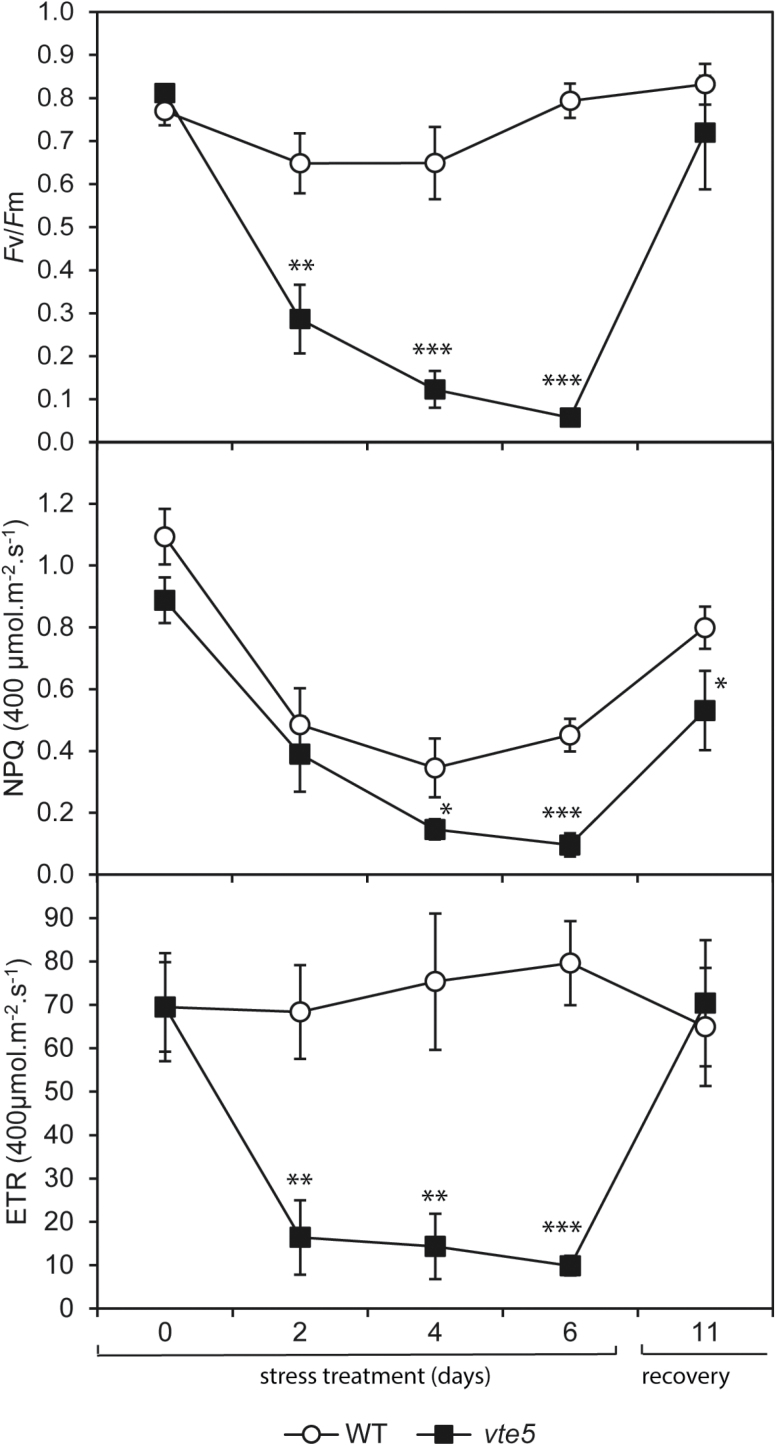
Photosynthetic parameters in WT and *vte5* over the time course of exposure to the combination of HT+HL stress. The photochemical efficiency of photosystem II (*F*v/*F*m), electron transport rate (ETR), and non-photochemical quenching (NPQ) values are the means ±SE of at least four biological replicates from plants exposed to HT+HL (820 µmol m^−2^ s^−1^, 16/8 h light/dark, 38/30 °C) for 6 days followed by 5 days of recovery at control light and temperature up to day 11. Asterisks indicate significant differences between WT and *vte5* (one-way ANOVA, followed by Holm-Sidak *post hoc* test): *P<0.05, **P<0.01, ***P<0.001.

To determine the effects of HL, HT, and HT+HL stress on photosynthetic activity, photosynthetic parameters were determined by measuring chlorophyll fluorescence (Supplementary Fig. S2). After 6 days of stress, WT and *vte5* plants were allowed to recover for 5 days under control conditions. The control plants were permanently grown under control conditions. Significant differences in the photosynthetic parameters were observed under HT+HL when comparing the two genotypes (Fig. 3). In *vte5* a strong and increasing reduction of the photochemical efficiency of PSII (*F*v/*F*m) was observed, resulting in almost complete photoinhibition after 6 days. The *vte5* plants reached close to normal values after 5 days of recovery under control conditions (Fig. 3, top panel). In WT, *F*v/*F*m decreased slightly under HT+HL but recovered while still under the combined stress. Both WT and *vte5* exhibited a significant reduction of NPQ under HL+HT. However, NPQ in WT began to recover at day 6 of HT+HL, whereas in *vte5* this parameter improved only during the 5-day recovery period under control conditions. ETR in WT under the HT+HL treatment remained almost constant throughout the stress treatment and recovery period. In contrast, HT+HL provoked a rapid and severe drop in ETR in *vte5,* with a recovery to control values after a 5-day recovery period.

### Plastoglobules accumulate under HT+HL in *vte5*

**Fig. 4.**
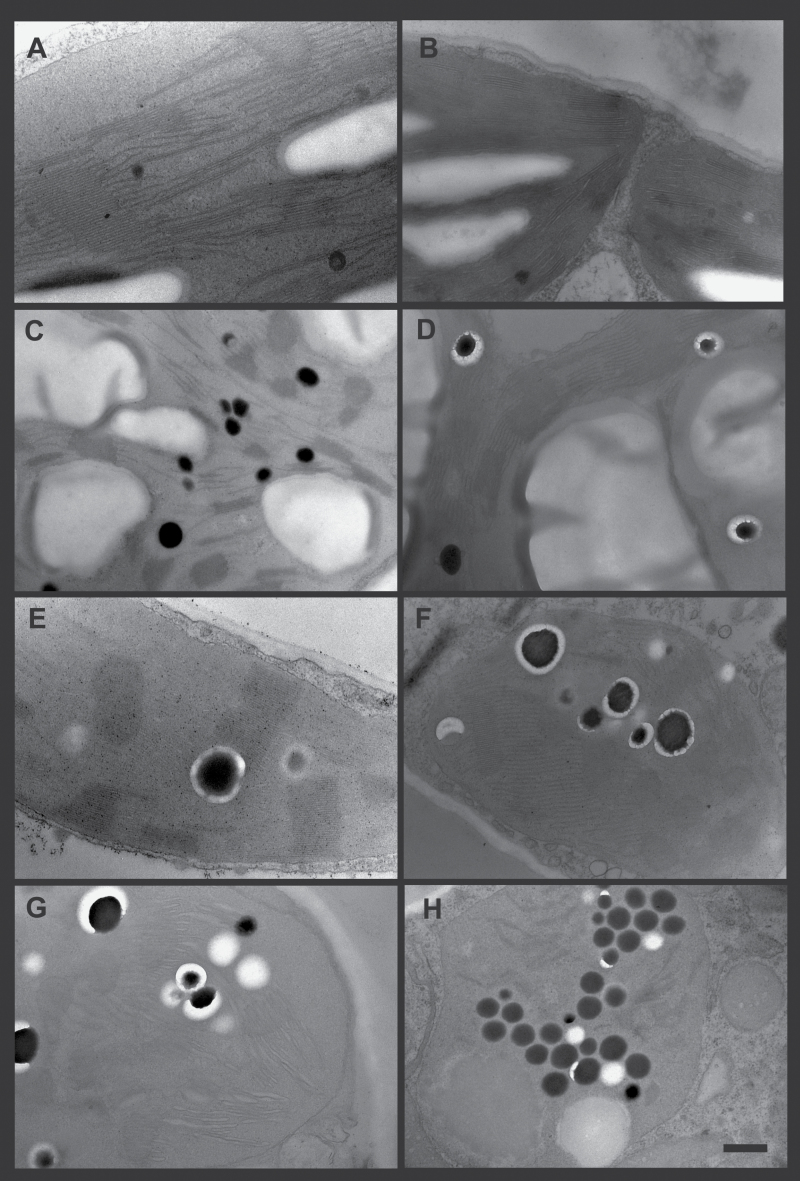
Changes in chloroplast ultrastructure under the control condition and HL, HT, and combined HT+HL stress. Transmission electron micrographs of WT (A, C, E, G) and *vte5* (B, D, F, H) leaves after 6 days of control (A, B), HL (C, D), HT (E, F), and combined HL+HT(G, H) treatments. Bar=500 nm.

Electron microscopy was carried out on leaf sections of plants exposed to control, HL, HT, and HT+HL conditions to determine the impact of the different conditions on chloroplast ultrastructure (Fig. 4 shows representative images). Under control conditions, WT (Fig. 4A) and *vte5* (Fig. 4B) chloroplasts were very similar. Under HL conditions, chloroplasts in both WT and *vte5* contained large starch granules (Fig. 4C and D) and larger plastoglobules also appeared, particularly in *vte5*. The *vte5* plastoglobules were surrounded by a non-osmiophilic ring appearing to contain additional, smaller globular structures (Fig. 4D). Under HT treatment, highly stacked thylakoids were observed and large plastoglobules surrounded by non-osmiophilic rings appeared in both WT and *vte5* (Fig. 4E and F). Under HL+HT, WT chloroplasts contained large plastoglobules with non-osmiophilic rings, and thylakoids appeared disorganized and swollen (Fig. 4G). *vte5* chloroplasts under HT+HL contained diminished and scattered thylakoids, increased numbers of plastoglobules (mostly without non-osmiophilic rings), as well as areas that contained amorphous material (Fig. 4H).

### Stress treatments change prenylquinone and carotenoid metabolism

**Fig. 5.**
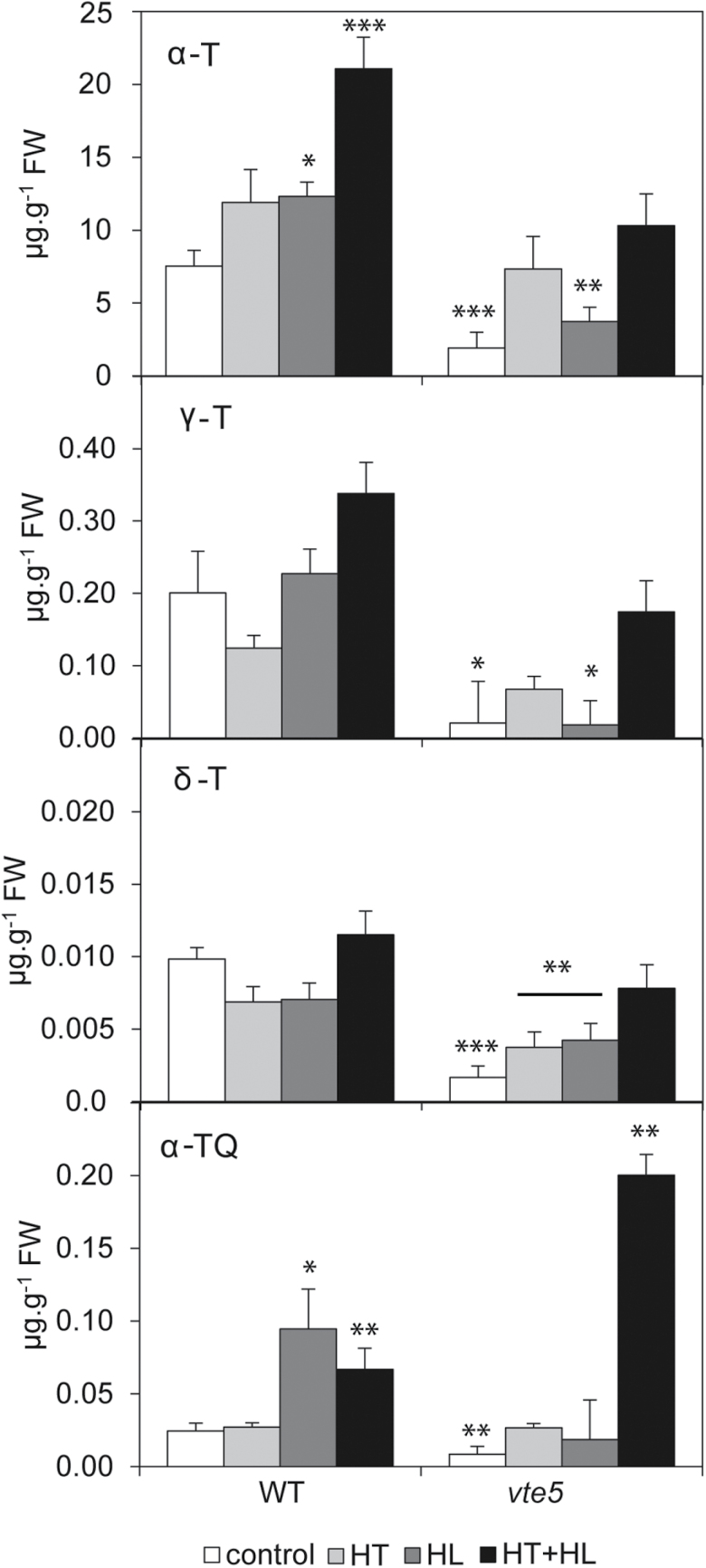
α-tocopherol (α-T), γ-tocopherol( γ-T), δ-tocopherol (δ-T), and α-tocopherolquinone (α-TQ) content in WT and *vte5* after exposure to control, HL, HT, and combined HT+HL stresses. Lipids were extracted from WT and *vte5* plants subjected to control or stress conditions for 6 days; lipids were analyzed by UHPLC-APCI-QTOF-MS. Values are the means ±SE of at least four biological replicates. Asterisks indicate significant differences between the WT (control) and *vte5* under the various the stress treatments (one-way ANOVA, followed by Holm-Sidak *post hoc* test): *P<0.05, **P<0.01, ***P<0.001.

Knockdown of *vte5* results in tocopherol deficiency (Almeida *et al.*, 2016). Under control conditions, the α-tocopherol content in *vte5* was one-third of that in WT, and other tocopherols displayed similar ratios (Fig. 5). Under both HL and HT stress, a 1.6-fold increase in α-tocopherol content was observed in WT. When exposed to the combination of HT+HL stress, WT plants showed a striking 2.8-fold increase in α-tocopherol. This indicates that HL and HT have additive effects on α-tocopherol accumulation in WT. In *vte5*, α-tocopherol also increased in an additive manner under the HT+HL condition, but only to around half the value observed in WT (Fig. 5). In addition, *vte5* showed a 3-fold increase in α-tocopherolquinone (α-TQ) when compared with WT under the HT+HL stress condition (Fig. 5).

**Fig. 6.**
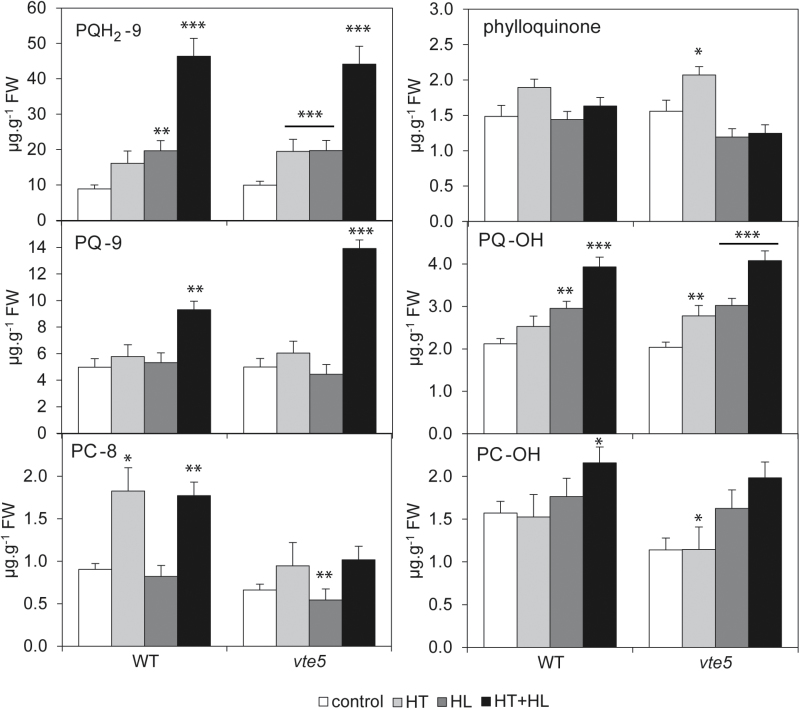
Plastoquinone, plastoquinone-derived quinones, and phylloquinone content in WT and *vte5* after exposure to control, HL, HT, and combined HT+HL stresses. Lipids were extracted from WT and *vte5* plants subjected to control or stress conditions for 6 days; lipids were analyzed by UHPLC-APCI-QTOF-MS. Values are the means±SE of at least four biological replicates. Asterisks indicate significant differences between the WT (control) and *vte5* under the various stress treatments (one-way ANOVA, followed by Holm-Sidak *post hoc* test): *P<0.05, **P<0.01, ***P<0.001.

Plastoquinone [consisting of oxidized PQ-9 (plastoquinone) and reduced PQH_2_-9 (plastoquinol)] and its derivatives PQ-OH (hydroxy-plastoquinone), plastochromanol-8 (PC-8), and hydroxy-plastochromanol-8 (PC-OH) were also measured (Fig. 6). PQH_2_-9 and PQ-OH concentrations were similar in WT and *vte5* under all conditions. PQ-9 was similar in WT and *vte5* under control conditions and the single-stress conditions, but was increased 1.5-fold in *vte5* compared with WT under the combined HT+HL stress (Fig. 6). PC-8 was reduced by around half in *vte5* compared with WT under HT and HT+HL, while PC-OH was similar in WT and *vte5*. Phylloquinone concentrations were not strongly affected in *vte5* and WT under any of the conditions (Fig. 6).

No significant changes in β-carotene and violaxanthin+neoxanthin concentrations were observed under any conditions when comparing WT with *vte5* (Supplementary Fig. S3). However, lutein increased 1.5- and 1.3-fold under HT in WT and *vte5* with respect to control conditions.

### Fatty acid phytyl esters accumulate massively in the *vte5* mutant

**Fig. 7.**
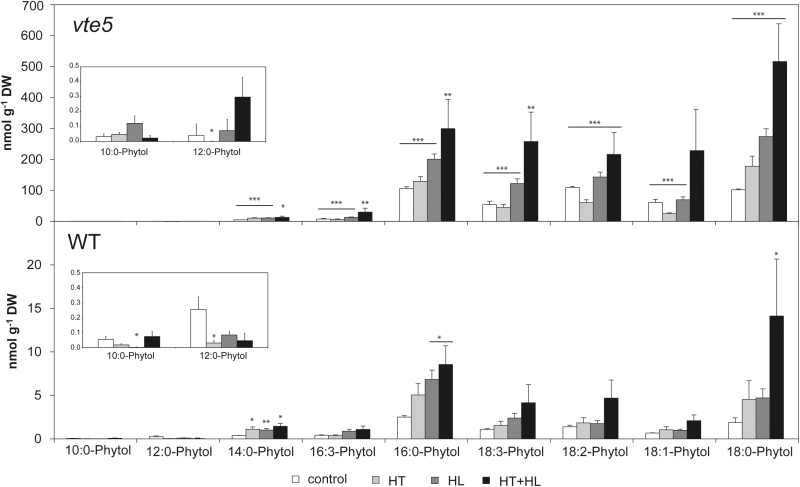
Fatty acid phytyl ester content in WT and *vte5* after exposure to control, HL, HT, and combined HT+HL stresses. WT and *vte5* were subjected to control or stress conditions for 6 days. Data represent the means±SE of at least three biological replicates. Asterisks indicate significant differences between the WT (control) and *vte5* under the various stress treatments (one-way ANOVA, followed by Holm-Sidak *post hoc* test) *P<0.05, **P<0.01, ***P<0.001.

Quantification of FAPEs indicated a striking accumulation under combined HT+HL stress in WT, but at far lower levels than in *vte5*. The most notable molecular species were 16:0-, 18:3-, 18:2-, 18:1-, and 18:0-phytol (Fig. 7). 18:0-phytol was the most abundant phytyl ester. In *vte5* its accumulation was 36-fold greater than in the WT exposed to HT+HL treatment.

### Free phytol levels increased under combined high-light and high-temperature stress

**Fig. 8.**
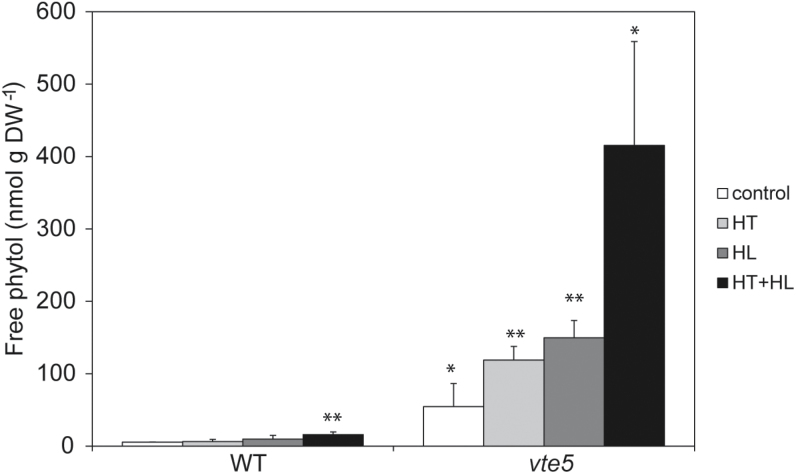
Free phytol content in WT and *vte5* after exposure to control, HL, HT, and combined HT+HL stresses. Data represent the means±SE of at least four biological replicates. Asterisks indicate significant differences between the WT (control) and *vte5* under the various stress treatments (one-way ANOVA, followed by Holm-Sidak *post hoc* test): *P<0.05, **P<0.01.

*vte5* plants accumulate free phytol in the leaf (Fig. 8) (Almeida *et al.*, 2016). When exposed to combined HT+HL, *vte5* plants had 7.6-fold higher concentrations than under control conditions. Even more strikingly, free phytol concentrations under HT+HL were 26-fold greater than in WT.

### Phytol toxicity assessed by electron transport activity

**Fig. 9.**
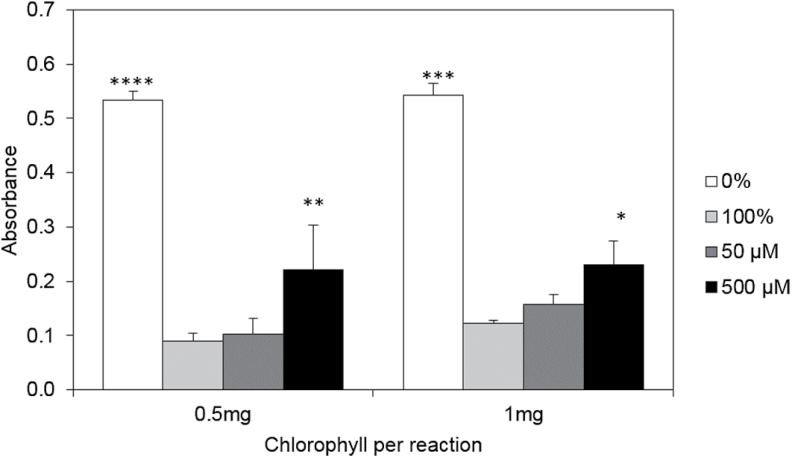
Assessment of phytol toxicity on photosynthetic electron transport in isolated thylakoid membranes. Photoreduction of DCPIP was measured in the presence of 50 and 500 μM phytol. 0% reduction refers to the negative control in the absence of both thylakoids and phytol. 100% refers to the Hill reaction in the absence of phytol. Absorbance was measured after 6 minutes of incubation. Data are the means±SE of three replicates. Asterisks indicate significant differences between assays at each time point (one-way ANOVA, followed by Holm-Sidak *post hoc* test): *P<0.05, **P<0.01, ***P<0.001, ****P<0.0001.

High free phytol concentrations were detected in *vte5* exposed to combined HT+HL. Phytol is presumed to be toxic due to its detergent-like structure and may therefore contribute to the photobleaching phenotype observed in *vte5* under HT+HL. To determine whether phytol may exert toxicity via perturbation of the thylakoid membrane and of photosynthetic electron transport, we carried out the Hill reaction in the presence of phytol. The Hill reaction is a method to measure photosynthetic electron transport in isolated thylakoid membranes (*P. sativum*) at the level of PSII (Fig. 9). In the Hill reaction, the synthetic electron acceptor DCPIP is reduced; this reaction can be measured spectrophotometrically. In the absence of phytol, DCPIP reduction was arbitrarily set to 100%; in a negative control experiment in the absence of thylakoids, DCPIP reduction did not occur and was set to 0%. The effects of the presence of two concentrations of phytol (50 µM and 500 µM) on the Hill reaction were measured. The phytol concentrations were calculated to be 50-fold and 500-fold those measured *in vivo* in *vte5* leaves under HT+HL. Two separate experiments were carried out using isolated thylakoids corresponding to either 0.5 mg or 1 mg chlorophyll per reaction. The Hill reaction was significantly inhibited only at 500 µM phytol.

### Gene expression profiles

To determine whether the observed biochemical changes could be due to altered gene expression under the HL, HT, and HT+HL conditions, transcript levels of genes encoding proteins involved in methyl-D-erythritol 4-phosphate, carotenoid, prenylquinone, chlorophyll, and phytol metabolism (Almeida *et al.*, 2016; Lira *et al.*, 2016) were measured by qPCR (Supplementary Fig. S4).

VTE5 showed a down-regulation of 76% in the *vte5* line under control conditions, close to the value previously published in Almeida *et al.* (2016). Under both the HL and HT conditions the expression patterns of genes encoding enzymes involved in tocochromanol, prenylquinone, chlorophyll degradation, carotenoid, and FAPE metabolic pathways (see Supplementary Fig. S4 for genes) were remarkably similar in both WT and *vte5*. Even under combined HT+HL stress, differences between WT and *vte5* were rather limited. However, some differences were observed: First, in WT as well as *vte5,* the abundance of the 1-deoxy-D-xylulose-5-P synthase (*DXS1*) transcript was diminished under HL and HT+HL. This potentially limits carbon supply to the plastidial isoprenoid pathway. However, this did not appear to be the case: with the exception of α-tocopherol in *vte5*, prenyllipids were unchanged or increased, particularly under the combined HT+HL stress. *NDC1* expression was remarkably up-regulated in *vte5* under HT+HL. In contrast, *VTE1* expression was lower in *vte5* than in WT under the same stress conditions. The low *VTE1* transcription level correlates with (i) with the accumulation of α-TQ, indicating insufficient activity in the redox recycling of α-TQ by VTE1, and (ii) the reduced levels of PC-8 that were observed in *vte5* (Fig. 6). In both WT and *vte5,* geranylgeranyl reductase (*GGDR*) mRNA abundance was diminished under all stress conditions except HL in WT, potentially provoking a reduction of chlorophyll synthesis. Higher expression of the plastoglobule-associated enzyme PES (phytol ester synthase) was observed to similar degrees under the HL and HL+HT conditions in both WT and *vte5*. However, only in *vte5* was a dramatic increase of FAPEs actually observed. This suggests that the transcriptional regulation had only a limited role in diverting the metabolic flux into the FAPE biosynthetic pathway.

## Discussion

Photosynthetic metabolism is among the primary processes that are strongly affected by environmental perturbations (Lichtenthaler and Burkart, 1999; Tezara *et al.*, 1999; Chaves *et al.*, 2009; Mathur *et al.*, 2014). Changes in temperature, in combination with other parameters such as high incident light intensity, constitute powerful challenges to plants. There is a compelling need to understand the mechanisms underlying plant adaptation to such multiple stresses connected to climate change. Only a few studies have investigated the impact of simultaneous HL and HT on the photosynthetic apparatus of plants (Qiu and Lu, 2003; Streb *et al.*, 2003; Quiles, 2006; Buchner *et al.*, 2015; Krause *et al.*, 2015; Gerganova *et al.*, 2016; Faik *et al.*, 2016).

Under combined HT+HL, adaptation of the photosynthetic machinery is mediated by changes in protein levels and remodeling of the thylakoid membrane (Burgos *et al.*, 2011; Szymanski *et al.*, 2014; Zhao *et al.*, 2016). This response includes not only membrane lipids but also lipophilic antioxidants, including tocopherols, plastoquinone, and plastochromanol. Both tocopherols and plastoquinone have been implicated in resistance to HL. A recent study that carefully dissected the distinct roles of tocopherols and plastoquinone indicated a predominant role for plastoquinone under HL (Ksas *et al.*, 2015). This was somewhat surprising, as plastoquinone is normally associated with electron transport and the role of a lipid antioxidant is usually attributed to tocopherols. Our experiments aimed to determine what makes plants (using tomato as the model system) tolerant to prolonged HL, HT, and combined HL+HT stress.

WT tomato plants exposed to HL, HT, or HT+HL stresses exhibited a striking increase in prenylquinone contents. In particular, α-tocopherol and plastoquinol were elevated. Compared with HT or HL alone, a near doubling of both compounds under combined HT+HL stress was observed, indicating an additive effect of the two stress conditions. The production of tocopherols has previously been correlated with oxidative stress (Havaux *et al.*, 2005; Kobayashi and DellaPenna, 2008). Our own recent work has suggested a correlation of the increase in α-tocopherol and plastoquinone with the normal functioning of the photosynthetic apparatus under HT stress (Spicher *et al.*, 2016). That study was, however, unable to resolve whether tocopherol or plastoquinone was the main contributor.

As a tool to investigate the role of tocopherol in protecting the photosystem, we used a line (*vte5*) in which phytol kinase (VTE5) is silenced. VTE5, which salvages phytol from chlorophyll degradation, contributes to ~70% of tocopherol biosynthesis. *vte5* plants were highly sensitive to combined HT+HL but not the single stresses; HT+HL resulted in extensive photobleaching (Fig. 2). This phenotype was likely brought about by photoinhibition, as was apparent from the severe reduction of *F*v/*F*m in *vte5* (Fig. 3). The tolerance of WT but not *vte5* plants to HT+HL suggested that tocopherol metabolism holds the key to HT+HL resistance in tomato.

When comparing the prenyllipid profiles of WT and *vte5* under HT+HL, tocopherols were diminished in *vte5*, in agreement with the defect in the tocopherol salvage pathway. In contrast, PQH_2_-9 (the reduced form of plastoquinone) was unchanged, and PQ-9 (the oxidized form of plastoquinone) was increased in *vte5*. This increase in PQ-9 is probably due to photoinhibition affecting PSII and its ability to reduce PQ-9. These findings provide strong evidence for the role of tocopherol rather than plastoquinone in tolerance to HT+HL stress. The amount of PC-8, the VTE1-dependent chromanol derivative of plastoquinone, was reduced under HT+HL. However, PC-8 is present at a far lower concentration than the oxidized and reduced forms of plastoquinone and α-tocopherol, and is therefore unlikely to contribute in a major way to HT+HL resistance. A possible explanation for the reduced concentration of PC-8 is the observed down-regulation of VTE1 in the *vte5* background. This down-regulation under HT+HL may also be responsible for the accumulation of α-TQ, an α-tocopherol oxidation product that depends on VTE1 for recycling to α-tocopherol (Eugeni Piller *et al.*, 2014). Moreover, this is additional evidence for the profound defect in tocopherol metabolism in *vte5*.

Why would tocopherols be particularly important for tolerance to combined HT+HL stress? Besides its antioxidant functions, α-tocopherol acts as a membrane component. It partitions into the hydrophobic phase of a membrane lipid bilayer and reduces membrane fluidity by physical stabilization of the membrane structure (Arora *et al.*, 2000; Wang and Quinn, 2000). Reduction of membrane fluidity may therefore contribute to the adaptation of the thylakoid membrane to HT. Although the membrane-stabilizing effect has not been shown in artificial membranes with thylakoid lipid composition, it is tempting to propose that the protective function under HT+HL lies in the combination of α-tocopherol’s antioxidant and membrane-stabilizing properties.

The inability of *vte5* to salvage phytol resulting from chlorophyll degradation leads to the accumulation of free phytol and FAPE biosynthesis (Lippold *et al.*, 2012; Almeida *et al.*, 2016). The FAPE content rose significantly in response to HL, HT, and HT+HL in both *vte5* and WT, but in a far more dramatic fashion in *vte5* (Fig. 7). FAPE synthesis is boosted by the high levels of free phytol that arise due to the increase in chlorophyll catabolism and simultaneous lack of *VTE5* activity. However, not only FAPEs increased; free phytol also reached much higher levels in *vte5* than in WT. Under HT+HL the levels were particularly high, and this can probably be attributed to increased chlorophyll catabolism under these photobleaching conditions.

Phytol is a long-chain alcohol which, similar to free fatty acids, may exert detergent-like effects that may be detrimental to membrane function (Sikkema *et al.*, 1995; Löbbecke and Cevc, 1995). In contrast, FAPEs (which lack a detergent-like structure) are not considered to be toxic and, in addition, they are sequestered in plastoglobules (Fig. 4) (Gaude *et al.*, 2007; Lippold *et al.*, 2012). Indeed, plastoglobule numbers visibly increased in *vte5* under HT+HL (Fig. 4). Free phytol may affect photosynthetic function and lead to photoinhibition and photobleaching in its own right. To determine the integrity of the photosynthetic electron transport chain, we carried out the Hill reaction in the presence of two concentrations of free phytol. Concentrations that were 500 times those measured in *vte5* under HT+HL only slightly reduced electron transport. These results strongly suggested that photoinhibition and photobleaching in *vte5* were not due to free phytol accumulation. Taken together, the evidence presented here indicates that tocopherols, more than any other of the metabolites analyzed, are responsible for tolerance to combined HT and HL stress in tomato.

## Supplementary data

Supplementary data are available at *JXB* online.

**Protocol S1**. PQ and PC purification.

**Table S1**. Primers used for each experiment.

**Fig. S1**. Phenotype of WT and *vte5* after 4 days of combined HL and HT.

**Fig. S2**. Chlorophyll fluorescence measurements.

**Fig S3**. Violaxanthin + neoxanthin, β-carotene, lutein in tomato leaves after exposure to control, HL, HT and combined HT+HL stress.

**Fig S4**. Expression of genes encoding isoprenoid metabolism-related enzymes.

Supplementary_protocols_Supplementary_Figures

## Author contributions

FK and LS designed the research. LS carried out the experimental work and statistical analysis. MR provided *vte5* line. LS, MR, and JA conducted gene expression analysis. KD and PD measured phytol and fatty acid phytyl esters. RP helped to prepare TEM cuts and observations. GG developed the UHPLC-APCI-QTOFMS method, conducted measurements, and identified compounds. LS and FK wrote the manuscript. All authors critically read the manuscript.

## References

[CIT0001] Almeida J, da Azevedo MS, Spicher L 2016 Down-regulation of tomato PHYTOL KINASE strongly impairs tocopherol biosynthesis and affects prenyllipid metabolism in an organ-specific manner. Journal of Experimental Botany 67, 919–934. 26596763 10.1093/jxb/erv504 PMC4737080

[CIT0002] Arora A, Byrem TM, Nair MG, Strasburg GM 2000 Modulation of liposomal membrane fluidity by flavonoids and isoflavonoids. Archives of Biochemistry and Biophysics 373, 102–109. 10620328 10.1006/abbi.1999.1525

[CIT0003] Buchner O, Stoll M, Karadar M, Kranner I, Neuner G 2015 Application of heat stress in situ demonstrates a protective role of irradiation on photosynthetic performance in alpine plants. Plant, Cell & Environment 38, 812–826. 10.1111/pce.12455 PMC4407927 25256247

[CIT0004] Burgos A, Szymanski J, Seiwert B, Degenkolbe T, Hannah MA, Giavalisco P, Willmitzer L 2011 Analysis of short-term changes in the *Arabidopsis thaliana* glycerolipidome in response to temperature and light. The Plant Journal 66, 656–668. 21309866 10.1111/j.1365-313X.2011.04531.x

[CIT0005] Chaves MM, Flexas J, Pinheiro C 2009 Photosynthesis under drought and salt stress: regulation mechanisms from whole plant to cell. Annals of Botany 103, 551–560. 18662937 10.1093/aob/mcn125 PMC2707345

[CIT0006] Collakova E, DellaPenna D 2003 Homogentisate phytyltransferase activity is limiting for tocopherol biosynthesis in Arabidopsis. Plant Physiology 131, 632–642. 12586887 10.1104/pp.015222 PMC166839

[CIT0007] Di Rienzo JA, Gonzalez LA, Tablada EM 2009 fg-Statistics: Statistical software for the analysis of microarray data Córdoba: RDNDA http://sites.google.com/site/fgStatistics/.

[CIT0008] Dörmann P 2007 Functional diversity of tocochromanols in plants. Planta 225, 269–276. 17115182 10.1007/s00425-006-0438-2

[CIT0009] Eugeni Piller L, Abraham M, Dörmann P, Kessler F, Besagni C 2012 Plastid lipid droplets at the crossroads of prenylquinone metabolism. Journal of Experimental Botany 63, 1609–1618. 22371323 10.1093/jxb/ers016

[CIT0010] Eugeni Piller L, Besagni C, Ksas B, Rumeau D, Brehelin C, Glauser G, Kessler F, Havaux M 2011 Chloroplast lipid droplet type II NAD(P)H quinone oxidoreductase is essential for prenylquinone metabolism and vitamin K1 accumulation. Proceedings of the National Academy of Sciences, USA 108, 14354–14359. 10.1073/pnas.1104790108 PMC3161580 21844348

[CIT0011] Eugeni Piller L, Glauser G, Kessler F, Besagni C 2014 Role of plastoglobules in metabolite repair in the tocopherol redox cycle. Frontiers in Plant Science 5, 298. 25018761 10.3389/fpls.2014.00298 PMC4071476

[CIT0012] Faik A, Popova AV, Velitchkova M 2016 Effects of long-term action of high temperature and high light on the activity and energy interaction of both photosystems in tomato plants. Photosynthetica 54, 611–619.

[CIT0013] Fitzpatrick LM, Keegstra K 2001 A method for isolating a high yield of Arabidopsis chloroplasts capable of efficient import of precursor proteins. The Plant Journal 27, 59–65. 11489183 10.1046/j.0960-7412.2001.01061.x

[CIT0014] Gaude N, Bréhélin C, Tischendorf G, Kessler F, Dörmann P 2007 Nitrogen deficiency in Arabidopsis affects galactolipid composition and gene expression and results in accumulation of fatty acid phytyl esters. The Plant Journal 49, 729–739. 17270009 10.1111/j.1365-313X.2006.02992.x

[CIT0015] Gerganova M, Popova AV, Stanoeva D, Velitchkova M 2016 Tomato plants acclimate better to elevated temperature and high light than to treatment with each factor separately. Plant Physiology and Biochemistry 104, 234–241. 27038602 10.1016/j.plaphy.2016.03.030

[CIT0016] Gruszka J, Kruk J 2007 RP-LC for determination of plastochromanol, tocotrienols and tocopherols in plant oils. Chromatographia 66, 909–913.

[CIT0017] Gruszka J, Pawlak A, Kruk J 2008 Tocochromanols, plastoquinol, and other biological prenyllipids as singlet oxygen quenchers—determination of singlet oxygen quenching rate constants and oxidation products. Free Radical Biology & Medicine 45, 920–928. 18634868 10.1016/j.freeradbiomed.2008.06.025

[CIT0018] Havaux M, Eymery F, Porfirova S, Rey P, Dörmann P 2005 Vitamin E protects against photoinhibition and photooxidative stress in *Arabidopsis thaliana*. The Plant Cell 17, 3451–3469. 16258032 10.1105/tpc.105.037036 PMC1315381

[CIT0019] Ischebeck T, Zbierzak AM, Kanwischer M, Dörmann P 2006 A salvage pathway for phytol metabolism in Arabidopsis. Journal of Biological Chemistry 281, 2470–2477. 16306049 10.1074/jbc.M509222200

[CIT0020] Kobayashi N, DellaPenna D 2008 Tocopherol metabolism, oxidation and recycling under high light stress in Arabidopsis. The Plant Journal 55, 607–618. 18452591 10.1111/j.1365-313X.2008.03539.x

[CIT0021] Krause GH, Winter K, Krause B, Virgo A 2015 Light-stimulated heat tolerance in leaves of two neotropical tree species, *Ficus insipida* and *Calophyllum longifolium*. Functional Plant Biology 42, 42–51. 10.1071/FP14095 32480652

[CIT0022] Ksas B, Becuwe N, Chevalier A, Havaux M 2015 Plant tolerance to excess light energy and photooxidative damage relies on plastoquinone biosynthesis. Scientific Reports 5, 10919. 26039552 10.1038/srep10919 PMC4454199

[CIT0023] Kummerová M, Krulová J, Zezulka S, Tríska J 2006 Evaluation of fluoranthene phytotoxicity in pea plants by Hill reaction and chlorophyll fluorescence. Chemosphere 65, 489–496. 16516947 10.1016/j.chemosphere.2006.01.052

[CIT0024] Kummerová M, Vánová L, Krulová J, Zezulka S 2008 The use of physiological characteristics for comparison of organic compounds phytotoxicity. Chemosphere 71, 2050–2059. 18336864 10.1016/j.chemosphere.2008.01.060

[CIT0025] Lichtenthaler HK, Burkart S 1999 Photosynthesis and high light stress. Bulgarian Journal of Plant Physiology 25, 3–16.

[CIT0026] Lippold F, vom Dorp K, Abraham M 2012 Fatty acid phytyl ester synthesis in chloroplasts of Arabidopsis. The Plant Cell 24, 2001–2014. 22623494 10.1105/tpc.112.095588 PMC3442583

[CIT0027] Lira BS, Rosado D, Almeida J, de Souza AP, Buckeridge MS, Purgatto E, Guyer L, Hörtensteiner S, Freschi L, Rossi M 2016 Pheophytinase knockdown impacts carbon metabolism and nutraceutical content under normal growth conditions in tomato. Plant & Cell Physiology 57, 642–653. 26880818 10.1093/pcp/pcw021

[CIT0028] Löbbecke L, Cevc G 1995 Effects of short-chain alcohols on the phase behavior and interdigitation of phosphatidylcholine bilayer membranes. Biochimica et Biophysica Acta 1237, 59–69. 7619843 10.1016/0005-2736(95)00076-f

[CIT0029] Loyola J, Verdugo I, González E, Casaretto JA, Ruiz-Lara S 2012 Plastidic isoprenoid biosynthesis in tomato: physiological and molecular analysis in genotypes resistant and sensitive to drought stress. Plant Biology 14, 149–156. 21974688 10.1111/j.1438-8677.2011.00465.x

[CIT0030] Maeda H, Song W, Sage TL, DellaPenna D 2006 Tocopherols play a crucial role in low-temperature adaptation and Phloem loading in Arabidopsis. The Plant Cell 18, 2710–2732. 17012603 10.1105/tpc.105.039404 PMC1626601

[CIT0031] Malferrari M, Francia F 2014 Isolation of plastoquinone from spinach by HPLC. Journal of Chromatography & Separation Techniques 5, 242.

[CIT0032] Martinis J, Glauser G, Valimareanu S, Kessler F 2013 A chloroplast ABC1-like kinase regulates vitamin E metabolism in Arabidopsis. Plant Physiology 162, 652–662. 23632854 10.1104/pp.113.218644 PMC3668060

[CIT0033] Martinis J, Kessler F, Glauser G 2011 A novel method for prenylquinone profiling in plant tissues by ultra-high pressure liquid chromatography-mass spectrometry. Plant Methods 7, 23. 21777468 10.1186/1746-4811-7-23 PMC3152938

[CIT0034] Mathur S, Agrawal D, Jajoo A 2014 Photosynthesis: response to high temperature stress. Journal of Photochemistry and Photobiology. B, Biology 137, 116–126. 10.1016/j.jphotobiol.2014.01.010 24796250

[CIT0035] Mène-Saffrané L, DellaPenna D 2010 Biosynthesis, regulation and functions of tocochromanols in plants. Plant Physiology and Biochemistry 48, 301–309. 20036132 10.1016/j.plaphy.2009.11.004

[CIT0036] Mène-Saffrané L, Jones AD, DellaPenna D 2010 Plastochromanol-8 and tocopherols are essential lipid-soluble antioxidants during seed desiccation and quiescence in Arabidopsis. Proceedings of the National Academy of Sciences, USA 107, 17815–17820. 10.1073/pnas.1006971107 PMC2955118 20837525

[CIT0037] Miret JA, Munné-Bosch S 2015 Redox signaling and stress tolerance in plants: a focus on vitamin E. Annals of the New York Academy of Sciences 1340, 29–38. 25586886 10.1111/nyas.12639

[CIT0038] Munné-Bosch S 2005 The role of alpha-tocopherol in plant stress tolerance. Journal of Plant Physiology 162, 743–748. 16008098 10.1016/j.jplph.2005.04.022

[CIT0039] Munné-Bosch S, Alegre L 2002 The function of tocopherols and tocotrienols in plants. Critical Reviews in Plant Sciences 21, 31–57.

[CIT0040] Nowicka B, Kruk J 2012 Plastoquinol is more active than α-tocopherol in singlet oxygen scavenging during high light stress of *Chlamydomonas reinhardtii*. Biochimica et Biophysica Acta 1817, 389–394. 22192719 10.1016/j.bbabio.2011.12.002

[CIT0041] Pfaffl MW, Horgan GW, Dempfle L 2002 Relative expression software tool (REST) for group-wise comparison and statistical analysis of relative expression results in real-time PCR. Nucleic Acids Research 30, e36. 11972351 10.1093/nar/30.9.e36 PMC113859

[CIT0042] Pretty J, Sutherland WJ, Ashby J 2010 The top 100 questions of importance to the future of global agriculture. International Journal of Agricultural Sustainability 8, 219–236.

[CIT0043] Qiu N, Lu C 2003 Enhanced tolerance of photosynthesis against high temperature damage in salt-adapted halophyte *Atriplex centralasiatica* plants. Plant, Cell and Environment 26, 1137–1145.

[CIT0044] Quadrana L, Almeida J, Otaiza SN 2013 Transcriptional regulation of tocopherol biosynthesis in tomato. Plant Molecular Biology 81, 309–325. 23247837 10.1007/s11103-012-0001-4

[CIT0045] Quiles MJ 2006 Stimulation of chlororespiration by heat and high light intensity in oat plants. Plant, Cell & Environment 29, 1463–1470. 10.1111/j.1365-3040.2006.01510.x 16898010

[CIT0046] Rastogi A, Yadav DK, Szymańska R, Kruk J, Sedlářová M, Pospíšil P 2014 Singlet oxygen scavenging activity of tocopherol and plastochromanol in *Arabidopsis thaliana*: relevance to photooxidative stress. Plant, Cell & Environment 37, 392–401. 10.1111/pce.12161 23848570

[CIT0047] Reynolds ES 1963 The use of lead citrate at high pH as an electron-opaque stain in electron microscopy. Journal of Cell Biology 17, 208–212. 13986422 10.1083/jcb.17.1.208 PMC2106263

[CIT0048] Sattler SE, Gilliland LU, Magallanes-Lundback M, Pollard M, DellaPenna D 2004 Vitamin E is essential for seed longevity and for preventing lipid peroxidation during germination. The Plant Cell 16, 1419–1432. 15155886 10.1105/tpc.021360 PMC490036

[CIT0049] Siger A, Kachlicki P, Czubiński J, Polcyn D, Dwiecki K, Nogala-Kalucka M 2014 Isolation and purification of plastochromanol-8 for HPLC quantitative determinations. European Journal of Lipid Science and Technology 116, 413–422.

[CIT0050] Sikkema J, de Bont JA, Poolman B 1995 Mechanisms of membrane toxicity of hydrocarbons. Microbiological Reviews 59, 201–222. 7603409 10.1128/mr.59.2.201-222.1995 PMC239360

[CIT0051] Smith MD, Schnell DJ, Fitzpatrick L, Keegstra K 2003 In vitro analysis of chloroplast protein import. Current Protocols in Cell Biology Chapter 11, Unit 11.16. doi: 10.1002/0471143030.cb1116s17. 10.1002/0471143030.cb1116s17 18228418

[CIT0052] Spicher L, Glauser G, Kessler F 2016 Lipid antioxidant and galactolipid remodeling under temperature stress in tomato plants. Frontiers in Plant Science 7, 167. 26925083 10.3389/fpls.2016.00167 PMC4756161

[CIT0053] Streb P, Aubert S, Bligny R 2003 High temperature effects on light sensitivity in the two high mountain plant species *Soldanella alpina* (L.) and *Rannunculus glacialis* (L.). Plant Biology 5, 432–440.

[CIT0054] Szymanski J, Brotman Y, Willmitzer L, Cuadros-Inostroza Á 2014 Linking gene expression and membrane lipid composition of Arabidopsis. The Plant Cell 26, 915–928. 24642935 10.1105/tpc.113.118919 PMC4001401

[CIT0055] Tezara W, Mitchell VJ, Driscoll SD, Lawlor DW 1999 Water stress inhibits plant photosynthesis by decreasing coupling factor and ATP. Nature 401, 914–917.

[CIT0056] Triantaphylidès C, Havaux M 2009 Singlet oxygen in plants: production, detoxification and signaling. Trends in Plant Science 14, 219–228. 19303348 10.1016/j.tplants.2009.01.008

[CIT0057] Valentin HE, Lincoln K, Moshiri F 2006 The Arabidopsis vitamin E pathway gene5-1 mutant reveals a critical role for phytol kinase in seed tocopherol biosynthesis. The Plant Cell 18, 212–224. 16361393 10.1105/tpc.105.037077 PMC1323494

[CIT0058] vom Dorp K, Hölzl G, Plohmann C, Eisenhut M, Abraham M, Weber AP, Hanson AD, Dörmann P 2015 Remobilization of phytol from chlorophyll degradation is essential for tocopherol synthesis and growth of Arabidopsis. The Plant Cell 27, 2846–2859. 26452599 10.1105/tpc.15.00395 PMC4682318

[CIT0059] Walther GR, Post E, Convey P, Menzel A, Parmesan C, Beebee TJ, Fromentin JM, Hoegh-Guldberg O, Bairlein F 2002 Ecological responses to recent climate change. Nature 416, 389–395. 11919621 10.1038/416389a

[CIT0060] Wang X, Quinn PJ 2000 The location and function of vitamin E in membranes (review). Molecular Membrane Biology 17, 143–156. 11128973 10.1080/09687680010000311

[CIT0061] Watson ML 1958 Staining of tissue sections for electron microscopy with heavy metals. Journal of Biophysical and Biochemical Cytology 4, 475–478. 13563554 10.1083/jcb.4.4.475 PMC2224499

[CIT0062] Zhao F, Zhang D, Zhao Y, Wang W, Yang H, Tai F, Li C, Hu X 2016 The difference of physiological and proteomic changes in Maize leaves adaptation to drought, heat, and combined both stresses. Frontiers in Plant Science 7, 1471. 27833614 10.3389/fpls.2016.01471 PMC5080359

