# Supplementary material for: Essential role for phytol kinase and tocopherol in tolerance to combined light and temperature stress in tomato
Source: J Exp Bot. 2017 Nov 24;68(21-22):5845–56. doi: 10.1093/jxb/erx356 (PMC5854125; doi:10.1093/jxb/erx356)
Supplement: Supplementary_protocols_Supplementary_Figures [file erx356_suppl_supplementary_protocols_supplementary_figures.pdf]

Supplementary material

Supplementary Protocol S1

PQ and PC purification. Plastoquinone (PQ) was isolated from about 200 g fresh spinach leaves according to a protocol derived from Malferrari and Francia (2014). Leaves were extracted in 1 L acetone under continuous stirring for 2 h followed by re-extraction in 300 mL. The extract was filtered using filter paper and the resulting solution was evaporated to dryness and reconstituted in 10 mL of petroleum ether. The concentrated extract was fractionated by open column chromatography on acidic alumina (Sigma-Aldrich) using increasing concentrations of diethylether in petroleum ether (0, 0.2, 2, 4, 8, 12, 16, 20, 24, 28% v/v). The 8% diethylether fraction containing PQ was evaporated, reconstituted in 0.5 mL THF:water (85:15, v/v) and injected in semi-preparative high performance liquid chromatography (HPLC) (see below).

Plastochromanol (PC-8) was purified from flaxseed oil using a protocol adapted from Siger *et al*. (2014). To 2 g of oil were added 0.5 g of pyrogallol, 20 mL ethanol and 2.5 mL KOH 60%. The mixture was heated at 80°C for 30 min, after which 50 mL of NaCl 1% was added. Once at room temperature, 50 ml of hexane:ethylacetate (9:1) were added and the resulting mixture was agitated for 30 min at 300 rpm. The two phases were then separated and the upper phase was washed twice with water. The organic phase was collected and dried with Na_2SO_4, filtered and evaporated to dryness. The dried extract was reconstituted in 10 mL petroleum ether and chromatographied as described above for plastoquinone. The 12% diethylether fraction containing plastochromanol was evaporated to dryness and reconstituted in 0.5 mL THF:water (85:15, v/v) for injection in semi-preparative HPLC.

Final purification of both PQ and PC-8 was achieved by reverse phase semi-preparative HPLC. The semi-preparative system was composed of a 1525 EF pump (Waters) and a dual wavelength UV detector (2487, Waters) equipped with a semi-preparative UV cell (path length 3 mm). An XTerra MS C18 column (19 x 150 mm, 5µm) thermostated at 60°C in a heated water bath was employed. Gradient conditions were as follows: solvent A was water and solvent B was methanol; initial conditions 90% B, 0-40.0 min 90-100% B, 40-77 min 100% B. Both prenyllipids were purified in one single injection of 500 µL. The flow rate was set to 8.0 mL/min. Detection was performed at 255 and 290 nm and fractions were collected every minute in 13x100 mm glass tubes using a Gilson FC203B fraction collector. An aliquot of the fractions corresponding to PQ and PC-8 was diluted 200-fold and re-injected in UHPLC-QTOFMS for confirmatory analysis. The remaining solutions were evaporated to dryness, yielding 2.0 mg of PQ and 1.2 mg of PC-8. The purity (>95%) of the standards was assessed by comparison of the signal intensities with those of authentic standards provided by Jerzy Kruk (Kruk, 1988; Gruszka and Kruk, 2007).

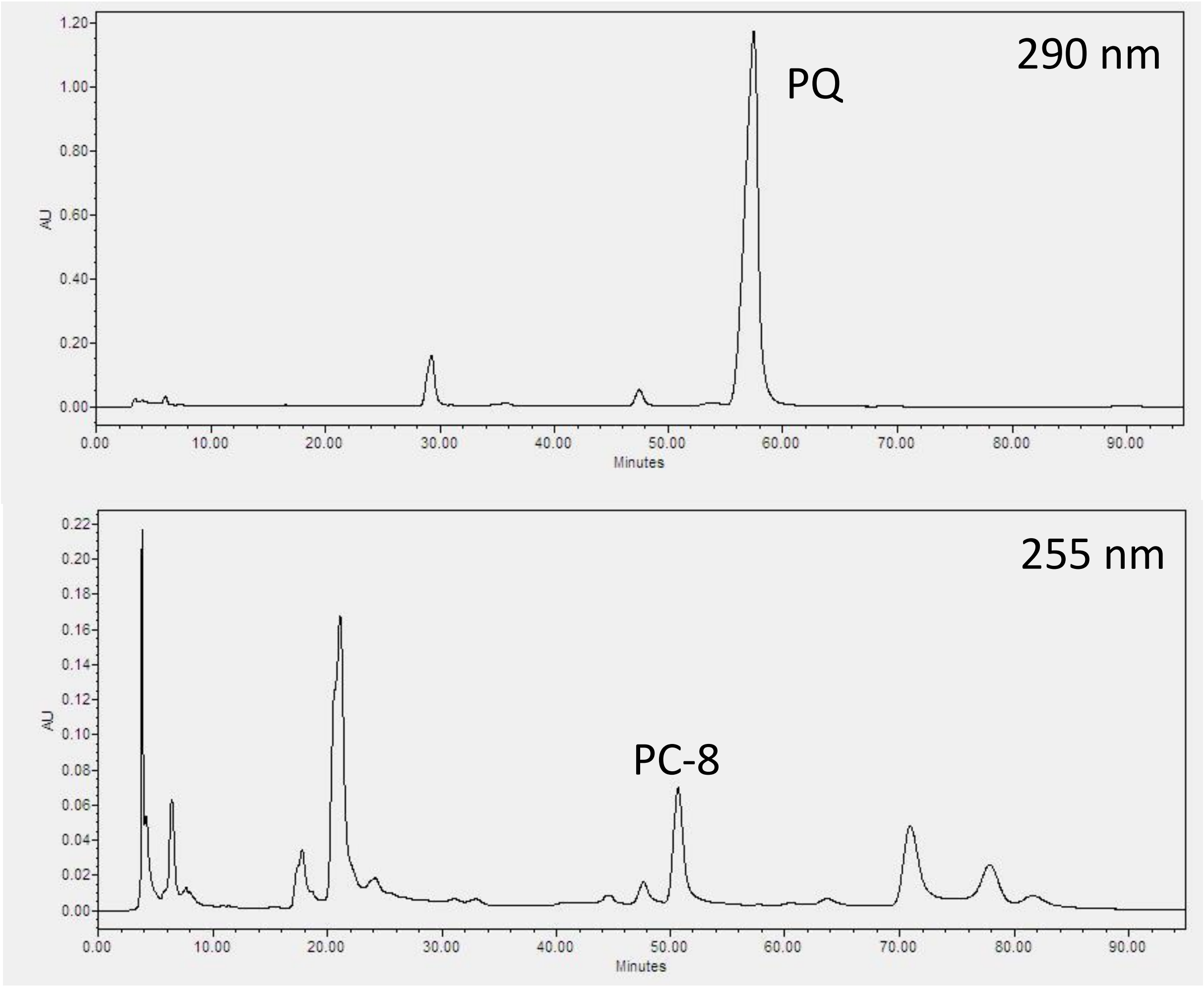

Chromatograms illustrating the final purification of plastoquinone (PQ) and plastochromanol-8 (PC-8) by preparative high performance liquid chromatography (HPLC).

Supplementary Table S1

Primers used for each experiment.

			Primer Sequence (5'-3')		Primer efficiency
Experiment	Primer name[a]	Tomato locus	Forward	Reverse	(fold increase)
qPCR	DXS(1)	Solyc01g067890	CAGGACTGGTGTGGTTTCAG	GGGATAGTTCACAGTGTCC	1.79
	GGDR	Solyc03g115980	CAGAGACGCTCGCTAAGG	GCTTCAGAGTCTGTCCGATATC	1.81
	HPPD(1)	Solyc07g045050	CCAGGGCAGGGGATATACTG	CTCCTTCCTCGTTTTTCAGC	1.89
	HPPD(2)	Solyc05g041200	CCAGGCGTGTGAAGAATTG	CGATCTAAACAGCTCAGAG	1.94
	VTE2	Solyc07g017770	CAATTCCAGTTCCTGCTGAG	CCTCCAACATGCTCTTGCGTG	1.81
	VTE3(1)	Solyc09g065730	CTTGACCAATCTCCTCATC	GCACGCCTTTCCTCCAGG	1.87
	VTE3(2)	Solyc03g005230	GCTAAGGCTAGGCAGAAGGAG	CAGGCAACCCCACCTATGG	1.86
	VTE1	Solyc08g068570	CGAACTCCTCATAGCGGGTATC	CACGCCAGTAAACCGAGGC	1.84
	VTE4	Solyc08g076360	CAGATCATCGTGCTGCTCAG	CCTCTCTGCTTGTACAGGAC	1.75
	VTE5	Solyc03g071720	CGTATCAGGACGGGCTCGC	TCACCACCACACATCATTGCTAATG	1.89
	VTE6	Solyc07g062180	AGCACAAGCATCAGTGTCTG	AAGAAAGCAGCCGCAATACC	1.92
	CHLG	Solyc09g014760	CCAATTCCTTCAGGTGCGGT	CCCACCAAGGCAAGCTGATA	1.88
	PPH	solyc01g088090	TATGGAGGGAGCAAGTACGC	TGGAGGGCAGAGGAAAAGTAC	1.91
	PAO	Solyc11g066440	TCAGAAGTGGGTGATATGGA	TATCCCCGTCATACACCTTA	1.87
	PPHL1/CLD1	Solyc02g070490	GATTTGGTGCTTCTGCCTTTC	GCTGTTTCTTCAGTTCCTTC	1.91
	HST	Solyc03g051810	GCTGCTAACTTGGTGCTC	GATCCTAGCACAGTCCCACG	1.74
	SPS	Solyc07g061990	GTGGTTGCGGATGACCTACTTA	CTTCTGTGATTTGTGGTGAGTTCC	1.86
	PSY(1)	Solyc03g031860	CGATGGTGCTTTGTCCGATAC	CTCATCAACCCAACCGTACC	1.91
	PSY(2)	Solyc02g081330	GCATCACACATAACTCCACAAGC	CGCATTCCTTCAACCATATCTCTG	1.89
	PDS	Solyc03g123760	CGTTCCGTGCTTCTCCGC	CTAGAACATCCCTTGCCTCCAG	1.90
	LCYβ	Solyc04g040190	GCACCCACATCAAAGCCAGAG	GCCACATGGAGAGTGGTGAAG	1.94
	PYP/PES(1)	Solyc01g098110	ACAGGACACAACTCCAACC	TAACCATCGCCATCTTCAGTG	1.95
	PES(2)	Solyc02g094430	CGAAGAGAGGGAAAAATGCCTGTG	GCTGCCATCCTGACAAATTCAGAC	1.87
	CAC	Solyc06g061150	CCTCCGTTGTGATGTAACTGG	ATTGGTGGAAAGTAACATCATCG	1.92
	EXPRESSED	Solyc07g025390	GCTAAGAACGCTGGACCTAATG	TGGGTGTGCCTTTCTGAATG	1.90
	NDC1	Solyc03g043750	TGCTGGTTGGAATTTGTGGG	CCATCAGTGAAACTTGGTGAAACAG	1.93
	CCD4A	Solyc08g075480	GTGGGGTAGTGAGTAGACATCC	ACGATCCCTGATAACTTAGGTGG	nc
	CCD4B	Solyc08g075490	GAAGACAGGAATGGTGAGC	CTATTACTTTTGGCATAGGACCC	1.93

[a] 1-deoxy-D-xylulose-5-P synthase (DXS); geranylgeranyl diphosphate reductase (GGDR); 4-hydroxyphenylpyruvate dioxygenase (HPPD); homogentisate phytyl transferase (VTE2); 2,3-dimethyl-5- phytylquinol methyltransferase (VTE3); tocopherol cyclase (VTE1); γ-tocopherol-C-methyl transferase (VTE4); phytol kinase (VTE5); chlorophyll synthase (CHLG); pheophytinase (PPH); pheophorbide a oxygenase (PAO); homogentisate solanesyl transferase (HST); solanesyl-diphosphate synthase (SPS); phytoene synthase (PSY); phytoene desaturase (PDS); chloroplast-specific β-lycopene cyclase (LCYβ); pale yellow petal/phytyl ester synthase (PYP/PES); clathrin adaptor complex medium subunit (CAC); naD(P)H dehydrogenase C1 (NDC1); carotenoid cleavage dioxygenase 4 (CCD4/ NCED4)

nc, not calculated due to the lack of expression in the analysed tissues

Supplementary Figure S1

Phenotype MT and vte5 after 4 days of combined HL and HT. Scale bar: 3cm.

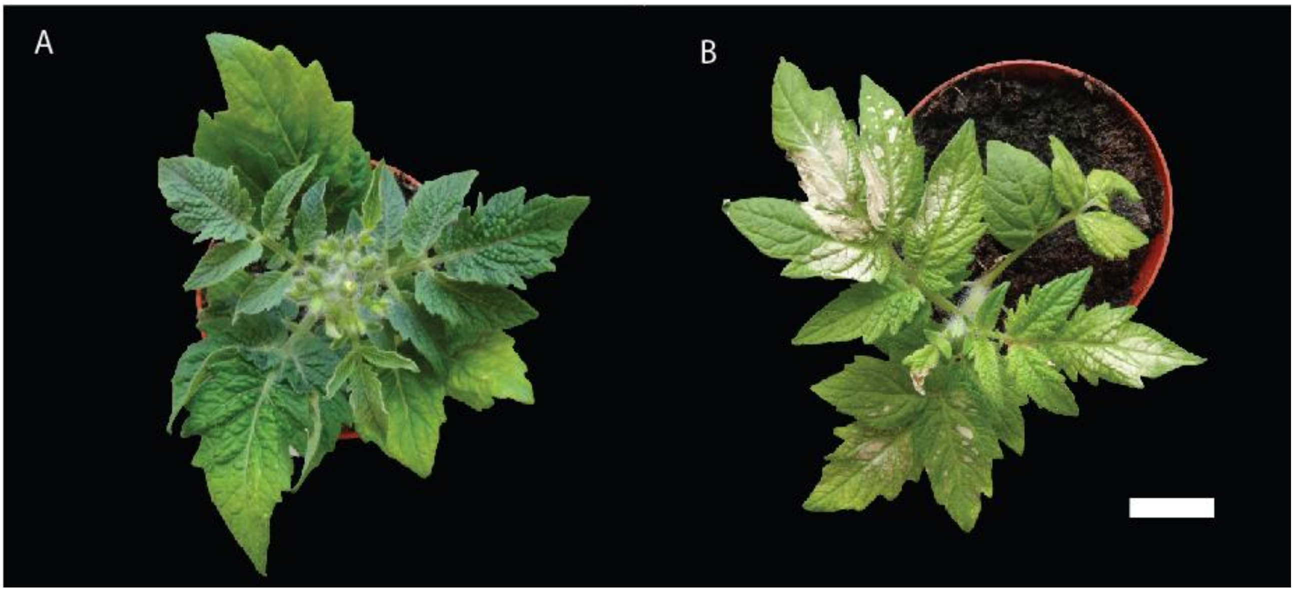

Supplementary Figure S2.

Chlorophyll fluorescence measurements. Photosynthetic efficiency in tomato leaves, WT and *vte5*, over a time course of exposure to different stress treatments. The ratio of maximum photochemical efficiency or optimum quantum yield of photosystem II (Fv/Fm), electron transport rate (ETR) and non-photochemical quenching (NPQ) values are the mean of at least 4 biological replicates (n = 4) from plants exposed to control conditions (250µmol.m^{-2}.s^{-1} of light, 16-h light/8-h dark, at 20/18°C), HT(250µmol.m^{-2}.s^{-1} of light, 16-h light/8-h dark, at 38/30°C), HL (820µmol.m^{-2}.s^{-}1, 16-h light/8-h dark, at 20/18°C), and combination of both stresses HT+HL (820µmol.m^{-2}.s^{-}1, 16-h light/8-h dark, at 38/30°C conditions for 6 days followed by 5 days of recovery at control temperature up to day 11. Means ± SE. The asterisks indicate significant differences between the control and the stress treatments for each time point (One-Way ANOVA, followed by Holm-Sidak, *posthoc* test, *, $p < 0.05$; **, $p < 0.01$; ***, $p < 0.001$).

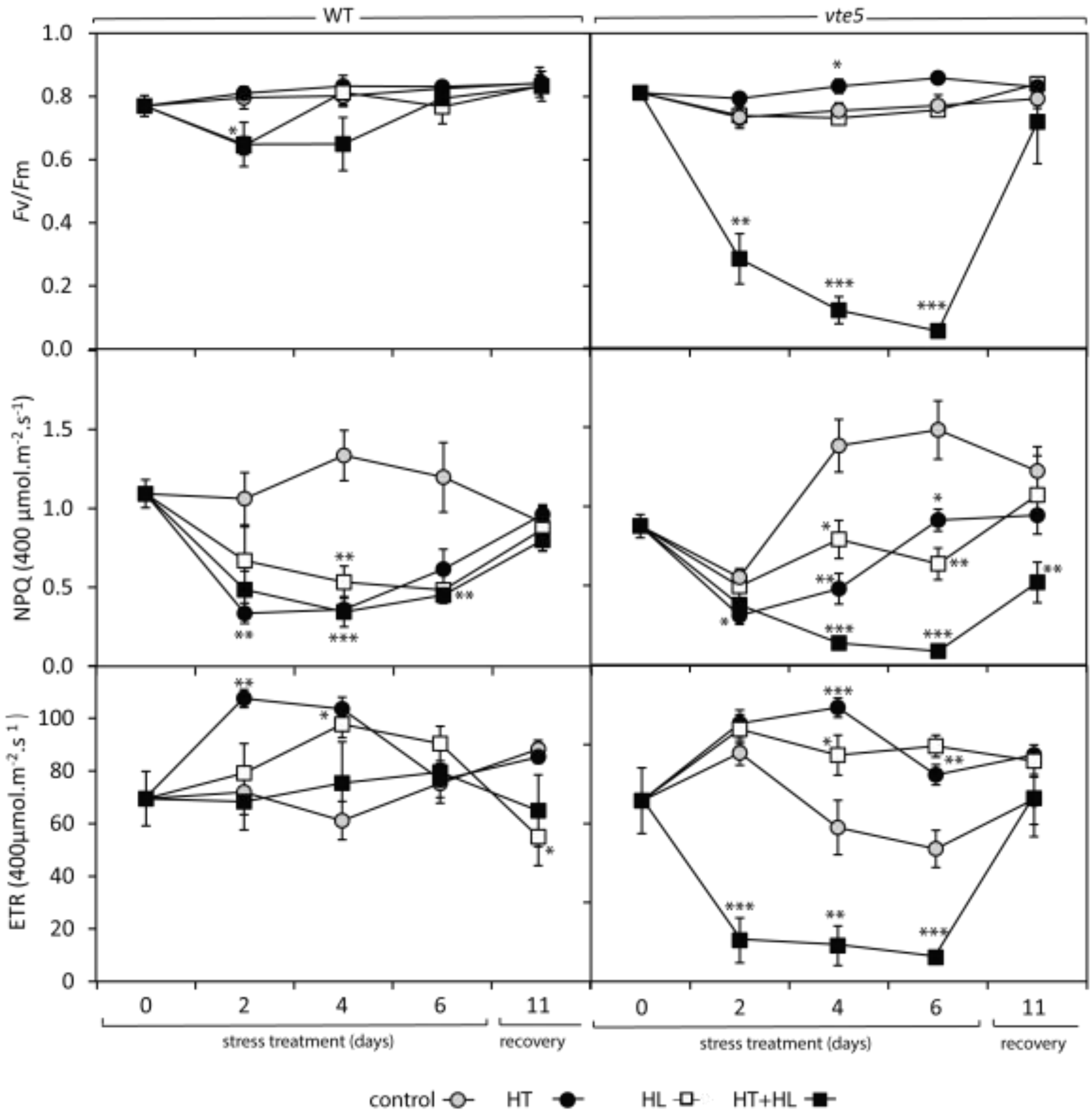
WT
vte5
Fv/Fm
NPQ (400 µmol.m-2.s-1)
ETR (400µmol.m-2.s-1)
stress treatment (days)
recovery
control
HT
HL
HT+HL

Supplementary Figure S3

Violaxanthin + neoxanthin, β-carotene, lutein in tomato leaves after exposure to control, HL, HT and combined HT+HL stress. Carotenoids were extracted from WT and *vte5* plants subjected to control or stress conditions for 6 days; Carotenoids were analyzed by UHPLC-QTOFMS. Values are means of at least 4 biological replicates ± SE. The asterisks indicate significant differences between the WT (control) and the stress treatments, including *vte5* (One-Way ANOVA, followed by Holm-Sidak, *posthoc* test, *, $p < 0.05$; **, $p < 0.01$; ***, $p < 0.001$).

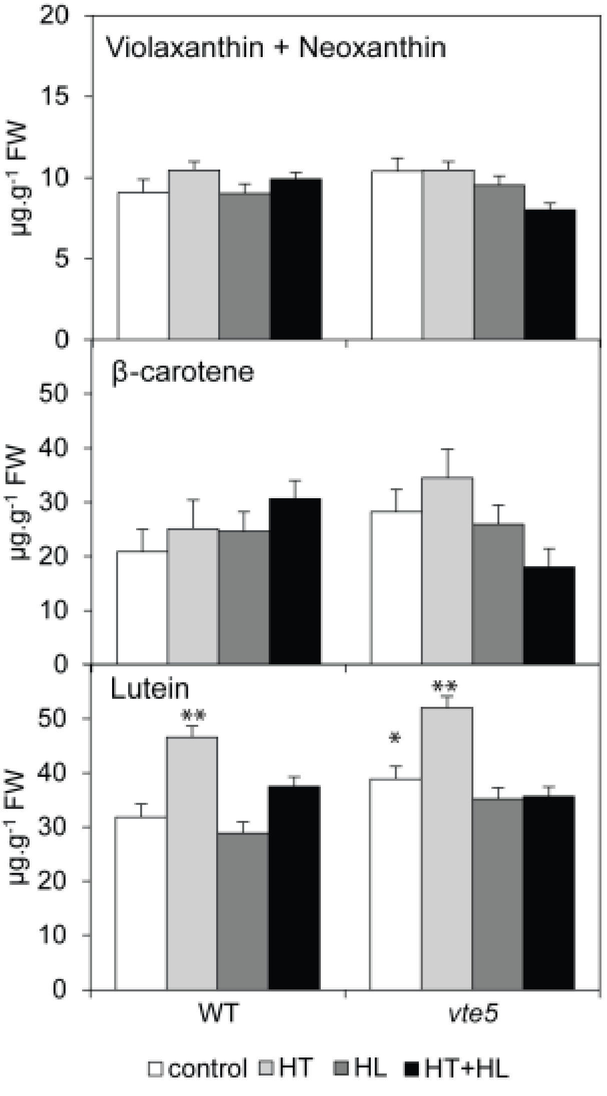

Supplementary Figure S4

Expression of genes encoding isoprenoid metabolism-related enzymes. The transcript abundance shown compares to WT control genotype of at least three biological replicates. The $\log_2$ of the expression ratios is indicated by a color scale where red and blue indicate statistically significant increases and decreases of transcript abundance ($p < 0.05$), respectively. 1-deoxy-D-xylulose-5-P synthase (DXS); geranylgeranyl diphosphate reductase (GGDR); tocopherol cyclase (VTE1); homogentisate phytyl transferase (VTE2); 2,3-methyl-5-phytylquinol methyltransferase (VTE3); γ-tocopherol-C-methyl transferase (VTE4); phytol kinase (VTE5); phytyl-phosphate kinase (VTE6); 4-hydroxyphenylpyruvate dioxygenase (HPPD); solanesyl-diphosphate synthase (SPS); homogentisate solanesyl transferase (HST); chlorophyll synthase (CHLG); chlorophyll dephytylase (CLD1/PPHL1); pheophytinase (PPH); pheophorbide *a* oxygenase (PAO); phytoene synthase (PSY); pale yellow petal/phytyl ester synthase (PYP/PES); phytoene desaturase (PDS); chloroplast-specific β-lycopene cyclase (LCYβ); naD(P)H dehydrogenase C1 (NDC1); carotenoid cleavage dioxygenase 4 (CCD4/ NCED4).

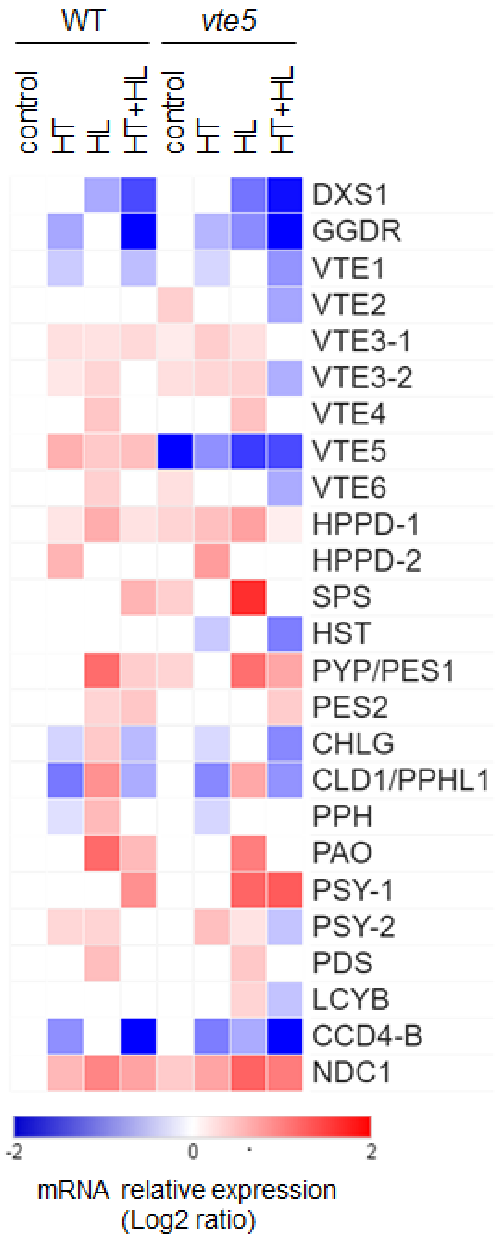
WT
vte5
control
HT
HL
HT+HL
control
HT
HL
HT+HL
DXS1
GGDR
VTE1
VTE2
VTE3-1
VTE3-2
VTE4
VTE5
VTE6
HPPD-1
HPPD-2
SPS
HST
PYP/PES1
PES2
CHLG
CLD1/PPHL1
PPH
PAO
PSY-1
PSY-2
PDS
LCYB
CCD4-B
NDC1
-2
0
2
mRNA relative expression
(Log2 ratio)
